# Effects of Finish Line Design and Fatigue Cyclic Loading on Phase Transformation of Zirconia Dental Ceramics: A Qualitative Micro-Raman Spectroscopic Analysis

**DOI:** 10.3390/ma12060863

**Published:** 2019-03-14

**Authors:** Roberto Sorrentino, Chiara Ottavia Navarra, Roberto Di Lenarda, Lorenzo Breschi, Fernando Zarone, Milena Cadenaro, Gianrico Spagnuolo

**Affiliations:** 1 Department of Neurosciences, Reproductive and Odontostomatological Sciences, Division of Prosthodontics and Digital Dentistry, University of Naples “Federico II”, via S. Pansini 5, 80131 Naples, Italy; zarone@unina.it (F.Z.); gspagnuo@unina.it (G.S.); 2 Department of Medical Sciences, University of Trieste, Piazza dell’Ospitale 1, 34129 Trieste, Italy; c.navarra@fmc.units.it (C.O.N.); rdilenarda@units.it (R.D.L.); mcadenaro@units.it (M.C.); 3 Department of Biomedical Science and Neuromotor Sciences, DIBINEM, University of Bologna, Via San Vitale 59, 40123 Bologna, Italy; lorenzo.breschi@unibo.it

**Keywords:** finish line, zirconia coping, Raman spectroscopy, transformation toughening, chewing simulation

## Abstract

Objectives: Stresses produced during the fabrication of copings and by chewing activity can induce a tetragonal-to-monoclinic (t–m) transformation of zirconia. As a consequence, in the m-phase, the material is not able to hinder possible cracks by the favorable mechanism known as “transformation toughening”. This study aimed at evaluating if different marginal preparations of zirconia copings can cause a premature phase transformation immediately after manufacturing milling and after chewing simulation. Methods: Ninety copings using three commercial zirconia ceramics (Nobel Procera Zirconia, Nobel Biocare Management AG; Lava Classic, 3M ESPE; Lava Plus, 3M ESPE) were prepared with deep-chamfer, slight-chamfer, or feather-edge finish lines (n = 10). Specimens were tested in a chewing simulator (CS-4.4, SD Mechatronik) under cyclic occlusal loads simulating one year of clinical service. Raman spectra were acquired and analyzed for each specimen along the finish lines and at the top of each coping before and after chewing simulation, respectively. Results: Raman analysis did not show any t–m transformation both before and after chewing simulation, as the typical monoclinic bands at 181 cm^−1^ and 192 cm^−1^ were not detected in any of the tested specimens. Conclusions: After a one-year simulation of chewing activity, irrespective of preparation geometry, zirconia copings did not show any sign of t–m transformation, either in the load application areas or at the margins. Consequently, manufacturing milling even in thin thickness did not cause any structural modification of zirconia ceramics “as received by manufacturers” both before and after chewing simulation.

## 1. Introduction

Due to patients increasing demand for aesthetics, as well as the introduction of the latest innovative dental technologies, the use of high-strength polycrystalline ceramics has become fairly widespread in the last decade [1,2,3,4,5,6]. All-ceramic materials, like zirconia, have to satisfy mechanical needs and provide good marginal adaptation and longevity comparable to traditional metal-ceramic prostheses [7,8]. Besides offering a more natural appearance of restorations, the biocompatibility and physical properties of zirconia frameworks are widely appreciated by clinicians for their enhanced chemical stability, high fracture toughness (K_IC_ = 9–10 MN/m^3/2^), and flexural strength (>1 GPa) [2,4]. 

Zirconia is an allotropic metastable material, which can be present in different crystallographic structures with the same chemical composition [9,10]. Structural changes occur by increasing the temperature:

$$\mathrm{Orthorombic}↔\mathrm{monoclinic}\overset{1170\;{}^{\circ}C}{\leftarrow \to }\mathrm{tetragonal}\overset{2680\;{}^{\circ}C}{\leftarrow \to }\mathrm{cubic}\overset{2370\;{}^{\circ}C}{\leftarrow \to }\mathrm{liquid}$$

**Figure 1:**
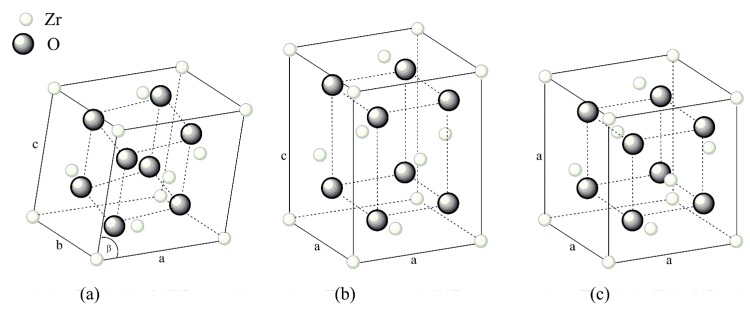
Schematic representation of polymorphisms of zirconia: (**a**) monoclinic, (**b**) tetragonal, and (**c**) cubic phase.

The transformations from cubic to tetragonal (c–t) and from tetragonal to monoclinic (t–m) under cooling are described as “martensitic”, (i.e., athermal and diffusionless); in the t–m transformation a volume expansion of about 5 vol.% occurs (when unconstrained) [11]. The three polymorphic phases, namely monoclinic (m), tetragonal (t), and cubic (c) of zirconia are represented in Figure 1.

To date, most zirconia restorations are fabricated by means of CAD/CAM (Computer-Aided Design/Computer-Aided Manufacturing) or copy-milling techniques [12]. During fabrication, they can be subjected to possible distortions, especially during sinterization and post-sintering cooling that, in particular, may negatively affect the marginal areas [13]. 

For zirconia core prostheses, the manufacturers recommend chamfer or round shoulder preparations, as they are considered to have the best precision, resistance, and aesthetics [8,14,15,16]. Nowadays, according to a minimally invasive approach, tooth preparations preserving sound tissues and preventing tooth weakening are requested. Providing a more acute marginal finish line, the feather-edge (or knife-edge) preparation was proposed as a less invasive alternative to chamfer [7,14,15]. Thin margins due to vertical preparation designs are likely affected by shrinkage and non-uniform distortions, resulting in inferior marginal adaptation and stress accumulation before clinical use [7,13,17], and increasing the susceptibility to clinical fractures [15]. Thus, they have not been extensively recommended in the clinical application of zirconia prostheses [4,13,18].

Framework fractures were reported as mechanical complications of zirconia restorations in several clinical studies [3,12,19,20]. Recent fractographic investigations showed that such fractures originated at the cervical margins of the frameworks [21,22]. Marginal design influenced the fracture resistance of zirconia restorations, resulting in highly divergent failure loads ranging from 450 to 1600 N [8,15,23,24,25], and the thinner the margins, the higher the risk of zirconia cracks [21,22].

The margin of a single crown framework is a key point where stresses accumulate [7,8,26]. It was hypothesized that stresses generated during CAD-CAM processes to grind the finishing lines of zirconia dental cores, as well as stresses induced by chewing activity, can induce a tetragonal-to-monoclinic transformation. The most important consequence of this t–m phase transformation, deleterious for the resistance of the material, would be that zirconia, in the m-phase, would not be able to hinder cracks that could arise by the favorable mechanism known as “transformation toughening” [2,10,27]. It was pointed out that the longevity of transformation-toughened zirconia was shorter when the material underwent cyclic loading [10,28].

Micro-Raman spectroscopy was used as a qualitative technique for the spectroscopic characterization of zirconia for biomedical applications. This technique was used to evaluate zirconia metastability, and visualize patterns of phase-transformation and related residual stresses on the surface of zirconia samples [29,30,31,32]. Furthermore, micro-Raman spectroscopy offers a non-destructive approach and enables us to obtain precise information about chemical composition through direct examination of specimens without compromising their integrity [33,34].

To date, there is little information on the integrity and mechanical reliability of zirconia cores with different marginal finish lines [4,7,8]. Thus, the present study aimed at evaluating if different marginal finish lines of zirconia crowns can cause premature aging of zirconia immediately after manufacturing and after chewing simulation using micro-Raman spectroscopy. The tested hypotheses were: (1) Different marginal preparations (i.e., deep-chamfer, slight-chamfer, feather-edge) do not produce a tetragonal-to-monoclinic phase transformation of zirconia cores; (2) a phase transformation does not occur at the margin of the samples prepared with different geometry after 1 year of fatigue load simulation.

## 2. Materials and Methods

### 2.1. Sample Preparation

**Figure 2:**
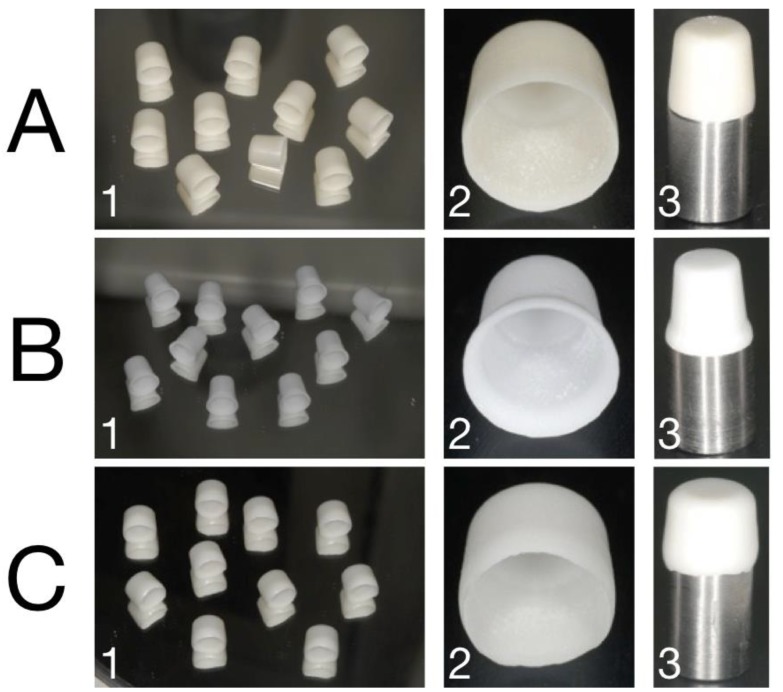
A1: Representative cluster of Group 1; A2: Detail of the slight-chamfer finish line; A3: Slight-chamfer zirconia coping on the proprietary metal abutment; B1: Representative cluster of Group 2; B2: Detail of the deep-chamfer finish line; B3: Deep-chamfer zirconia coping on the proprietary metal abutment; C1: Representative cluster of Group 3; C2: Detail of the knife-edge finish line; C3: Knife-edge zirconia coping on the proprietary metal abutment.

Three standardized stainless steel abutments [35,36,37] were designed in the shape of truncated cones on the basis of the average dimensions of a maxillary premolar with a height of 7 mm and a diameter of 8 mm [38] using a dedicated CAD software (Exocad, DentalCAD, Exocad GmbH, Darmstadt, Germany). Three different finish lines were used: Deep-chamfer (depth of 1 mm), slight-chamfer (depth of 0.5 mm), and feather-edge (no depth). All masters were designed and milled with a total occlusal convergence of 10 degrees [39] (Figure 2). Then, the abutments were physically fabricated using CAM technology by milling stainless steel cylinders in the designed shapes.

Three zirconia available on the market were selected: NobelProcera Zirconia, (Nobel Biocare Management AG, Zürich-Flughafen, English Switzerland), Lava Classic (3M ESPE, St. Paul, MN, USA), and Lava Plus (3M ESPE, St. Paul, MN, USA). Thirty zirconia copings for each material were prepared with a deep-chamfer, slight-chamfer, or feather-edge finish line (n = 10 per group). All copings had a thickness of 0.6 mm [40]. Groups and compositions are listed in Table 1.

### 2.2. Chewing Simulation

Zirconia copings were placed on their proprietary stainless steel abutments used for their preparation before impression. One drop of paraffin gel was used between the copings and the stainless steel abutments to avoid minor movements during the simulated chewing process.

**Figure 3:**
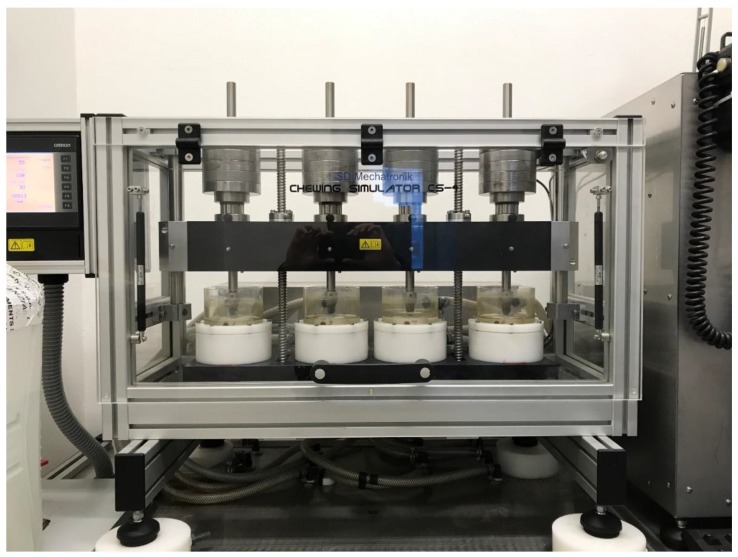
The chewing simulator (CS-4.4, SD Mechatronik).

**Figure 4:**
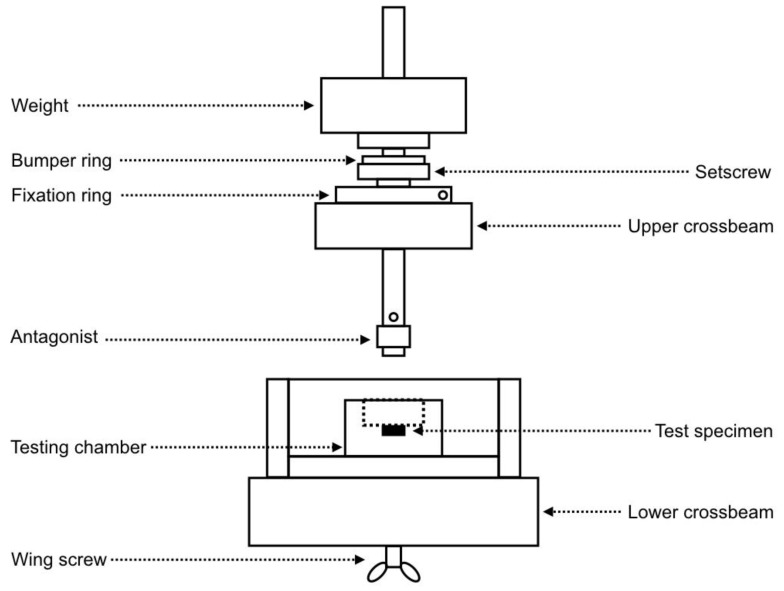
Schematic representation of the testing chamber of the CS-4.4 device.

A chewing simulator (CS) was used to simulate occlusal loading (CS-4.4, SD Mechatronik, Munich, Germany) (Figure 3). The device was made up of a sample holder and an antagonist (i.e., an aluminum cylinder of 8 mm in diameter) mounted on a crosshead with vertical motion (Figure 4). The crosshead was lifted by means of an endless screw and slowly sunk back to the surface of the specimen. As soon as the antagonist touched the surface, it detached from the crosshead and transferred the load of dead-weights to the sample surface. This mechanism ensured a no-impact and equal load for all specimens, which were mounted on a horizontal carrier. The occlusal loading device had four separated testing chambers that were fixed to the lower crosshead. In the present experimental setup, the antagonists were screwed to vertically adjusted carriers in force-supported vertical motion elements. These elements generated an occlusal load equal to 50 N for each specimen [41,42].

According to data reported in literature [41,42,43,44], test parameters were set as follows: Wet environment, 48 h with 1 Hz frequency for a total of 240,000 cycles with a downward and upward speed of 16 mm/s. These occlusal parameters simulated 1 year of clinical service [44].

### 2.3. Micro-Raman Analysis

A modular research spectrograph (Renishaw InVia; Renishaw plc, Gloucestershire, UK) connected to an optical microscope (Leica DM/LM optical microscope; Leica, Wetzlar, Germany) was used to investigate the crystalline configuration of zirconia. A near-infrared diode laser operating at 785 nm was used to induce the Raman scattering effect. The spectral coverage ranged from 100 to 3450 cm^−1^ with an average spectral resolution of 5 cm^−1^ [45]. Instrument calibration was determined before data acquisition by comparison with the spectrum of a single silicon crystal. Specimens were placed on a calcium fluoride glass under the optical microscope (Leica DM/LM optical microscope; Leica, Wetzlar, Germany) on a computer-controlled X-Y-Z stage, focusing the laser beam with 2 levels of magnification: 20× (laser power on the specimen surface ~40 mW), which provided the detection of monoclinic zirconia in a quite large area, and 100× (laser power on the specimen surface ~8 mW), to more precisely detect areas of presence of monoclinic zirconia.

Twenty spectra for each specimen were acquired, before and after chewing simulation, along the marginal finish line and 5 at the top of each coping, thus where the load was distributed and applied, respectively, to assess if phase-transition of the zirconia occurred. The spectral range was set between 100 and 700 cm^−1^ (zirconia fingerprint region), and the exposure time for each scan was 40 s. Acquired data were then analyzed with a spectrographic analysis software (Grams/AI 7.02; Thermo Galactic Industries Corp., Salem, NH, USA).

## 3. Results

**Figure 5:**
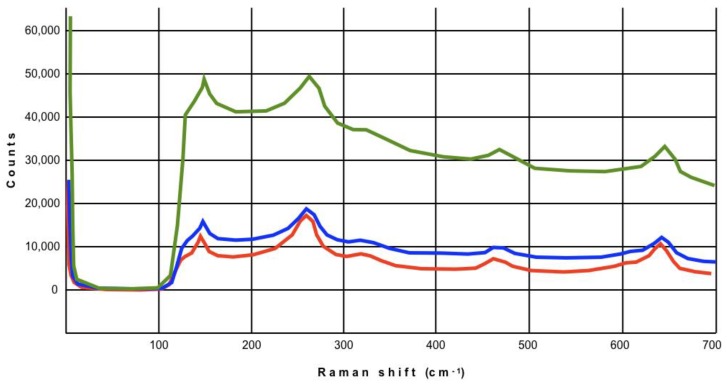
Representative Raman spectra obtained on slight-chamfer, deep-chamfer, and knife-edge finish lines. No monoclinic zirconia bands are visible (arrow indicates areas where they should occur).

None of the Raman spectra acquired at the margins and on the top of the copings showed the presence of monoclinic zirconia in the pre-chewing situation, irrespective of the different margin geometry. Moreover, when the spectra acquired before and after chewing simulation were compared, no changes were detected in any group, at both magnifications, as showed representatively in Figure 5. In fact, the spectral region between 100 and 300 cm^−1^ (containing all the vibrational bands needed to provide reliable information on the extent of the t–m transformation) did not show the typical bands of monoclinic zirconia at 181 cm^−1^ and 192 cm^−1^. 

No significant differences either in the shape of the spectra and in the height and width of the peaks were observed in all the tested specimens, both as received by manufacturers and after chewing simulation (Figure 5).

Micro-Raman spectroscopy was used to qualitatively identify possible crystallographic phase transformation of zirconia, but since no changes were detected in any spectra, no statistical analyses were performed.

## 4. Discussion

Zirconia is a restorative material widely used in dentistry and extensively tested for mechanical properties [46], fracture load [47], bond strength [48], cell proliferation [49], and translucency [10,50]. To date, in the authors’ knowledge, in the literature there are no reports that studied the top and the finish lines of zirconia copings with Raman spectroscopy, evaluating crystallographic changes soon after manufacturing milling and after chewing simulation. Consequently, the aim of the present study was to investigate the crystallographic changes of zirconia at the top and along the prosthetic margins of zirconia copings after 1 year of simulated clinical service. Particularly, both aging and fatigue were tested; the multifactorial phenomenon of aging was proven to be influenced not only by temperature, wetness, and hydrothermal stress, but also by surface defects, mechanical stress, and processing techniques [2,10,31,51,52]. The fabrication processes could influence these aspects, whereas dynamic chewing simulation was used to evaluate the possible influence of fatigue loads.

The present investigation was not conceived as a load-to-fracture study but as a qualitative evaluation of the possible phase transformation induced only by fabrication processes of zirconia copings as received by manufacturers. As the micro-Raman analyses were performed not only on the occlusal surfaces but also at the level of the prosthetic margins, it was necessary to remove the copings from the abutments so as to analyze the inner part of the finish lines. The use of conventional cements would have made such analyses impossible, so it was decided that a stabilizing medium would be used to avoid minor movements during the dynamic load of the copings. Indeed, the present investigation was designed to include the minimum possible number of study variables so as to detect the influence of milling procedures on possible zirconia phase transformation solely.

The results of the Raman spectroscopic analyses performed in this investigation showed that the machining processes needed to obtain the complex framework crown shaping did not generate the phenomenon of “transformation toughening”, either on the top or at the margins of the restorations. Similarly, specimens that underwent an in vitro occlusal loading challenge (simulating one year of clinical service) showed monoclinic zirconia neither on the top of the dental framework (where the load was applied) nor at the margins. Thus, both the experimental hypotheses tested were accepted since (1) different marginal preparations (i.e., deep-chamfer, slight-chamfer, feather-edge) did not produce a t–m phase transformation of zirconia cores and (2) phase transformation did not occur on specimens prepared with different prosthetic geometries after 1 year of fatigue load simulation. These results are remarkable since it was reported that zirconia under wear can show up to 40% vol of m-zirconia [28], so the absence of this phase in the CAD-CAM prepared cores can be considered a good starting point to support a long-lasting durability of zirconia dental prostheses.

Zirconia for dental use is available in three formulations: (1) dispersion-toughened ceramic, in which t-zirconia particles are dispersed in alumina (zirconia toughened alumina, ZTA); (2) mullite (zirconia toughened mullite, ZTM) as partially stabilized zirconia (PSZ), in which zirconia is stabilized in the t and c phase by addiction of dopants (yttrium, magnesium, etc.); (3) tetragonal zirconia polycrystals (TZP) that contain almost 98% of t-zirconia grains, which are so fine to need only a small amount of dopants (3 mol% of yttrium oxide) [39]. Among the materials tested in this study, Nobel Procera Zirconia is an yttrium-partially stabilized zirconia (Y-PSZ) based on very fine zirconium grains (0.3–0.5 μm) partially stabilized by the addition of 4.5%–5.5% yttrium oxide (Y_2_O_3_), as disclaimed by manufacturers; LAVA Classic and LAVA Plus are single-phase tetragonal zirconia polycrystals doped with yttrium (Y-TZP) as deduced by their composition. 

Thanks to the presence of dopants, during the processing of zirconia frameworks or monolithic crowns, zirconia polycrystals remain in the tetragonal polymorph phase (TZP), metastably retained when the temperature decreases [53]. Such a metastable tetragonal phase offers an interesting behavior that makes it mechanically more resistant than the monoclinic one: When a crack starts at the tetragonal zirconia surface, the tensile stress concentration induces the transformation of the grains nearby the crack from metastable t-ZrO_2_ to m-ZrO_2_, the monoclinic crystals being larger than the tetragonal ones. In fact, the energy dissipation mechanism determines a 3%–5% volume increase of the crystals, constrained by the surrounding ones, resulting in a favorable compressive stress that acts as a crack limiter. Such a stress-induced t–m phase transformation of zirconia crystals under load is known as “phase transformation toughening” (PTT) and remarkably increases the fracture toughness and the flexural strength of the material, much higher than in other ceramics for dental use [2,10,15,21]. At room temperature, the transformation from tetragonal to monoclinic is a one-way process. This means that after the t–m transformation has occurred, zirconia cannot exhibit the phase transformation toughening and its crack-hindering effect [51,52,54]. Nevertheless, occasionally a spontaneous, slow transformation of the crystals from the tetragonal to the monoclinic phase in the absence of any mechanical stress has been reported to occur over time (so-called “low temperature degradation”, LTD), decreasing the mechanical properties of the material and exposing zirconia frameworks at the risk of spontaneous catastrophic failures [10,51]. This phenomenon is accelerated by several factors, such as wetness, grain size, temperature, vapor, surface defects of the material, type, percentage, distribution of stabilizing oxides, and processing techniques [10,31,55] and can be deleterious especially if it occurs at the margins of the prosthetic restorations. 

Previous studies used X-ray diffractometry (XRD) to detect the crystallographic changes of zirconia but this technique is limited by its inability to detect the monoclinic zirconia if its content is lower than 5%, making it unsuitable to identify the start of the transformation [56,57]. For this reason, the more sensitive Raman spectroscopy was used in the present study. Raman spectroscopy is based on the change of wavelength of light that occurs if a light beam is deflected by molecules. If a beam of light (in our case a 785 nm near-infrared diode laser) hits the surface of a specimen, a small amount of light is rejected in a direction different from the incident one. An exiguous part of this scattered light changes its wavelength (i.e., Raman effect), providing vibrational information of the chemical nature of the specimen. Raman micro-spectroscopy can easily detect the presence of monoclinic zirconia, because its bands are clearly visible at 181 cm^−1^ and 192 cm^−1^ [58]. In a recent systematic review by Pereira et al. about low-temperature degradation of Y-TZP ceramic, the authors affirmed that the Raman technique is one of the most powerful tools for characterizing zirconia LTD [59]. 

Current manufacturers’ recommendations and literature suggest that shoulder, chamfer, or slight-chamfer marginal finish lines should be prepared for zirconia restorations [13,60], especially if the convergence angle of the tooth abutment is increased [61]. These preparations transfer a minimum of masticatory stresses from the coping to the veneering porcelain [62]. On the other hand, a knife-edge finishing line provides a minimally invasive approach, especially in vital anterior teeth, inclined teeth, etc. [7]. Crown tooth preparations without a defined finish line were historically defined in several ways, such as knife-edge, feather-edge, or shoulderless [7]. Generally, they may be termed vertical preparations as opposed to horizontal ones (shoulder, chamfer) [18,63]. These tooth preparations require an acute, knife-edge margin of the restoration and their most common indication has been the use of periodontally involved teeth as abutment for fixed prostheses [7,18,63].

Previous in vitro studies focused on the role of finish line preparation in determining marginal adaptation and fracture load resistance of zirconia crowns [13,15,18,64,65]. Komine et al. reported no differences in marginal adaptation if zirconia copings were prepared with shoulder, rounded shoulder, or chamfer [60], while a better marginal adaptation of zirconia crowns with feather-edge finish line compared to those with chamfer, shoulder, and mini-chamfer finish line types was reported by Comlekoglu et al. [13]. Nevertheless, authors did not recommend the application of the feather-edge finish line for clinical use [13], since it could lead to a wedging effect at the margins [66]. An in vitro study found that the finishing line design did not influence the fracture resistance of veneered zirconia restorations [26], as partially confirmed by Beuer et al., who found no differences in fracture load resistance between shoulderless and shoulder preparation of zirconia copings [64]. On the contrary, Reich at al. found that in Y-TZP zirconia crowns, feather-edge preparations showed a 38% increase in fracture load compared to chamfer preparations, regardless of the coping thickness [15]. Most of these fracture strength studies were conducted under controlled static stress to induce the fracture of the specimens and were only observational, not considering the structural changes that can occur in zirconia, while the present study evaluated the crystallographic changes of zirconia copings prepared with a deep-chamfer, slight-chamfer, or feather-edge finish line after a dynamic load. The fact that no changes were detected may be explained by the observation that zirconia crowns can survive 3.5 million loading cycles simulating seven years of function before fracture [26] and that the predicted failure probability of monolithic zirconia crowns in five years is 0.002 [67]. Future laboratory studies and randomized clinical trials (RCTs) may clarify development of the zirconia structural changes after prolonged clinical service to assess the longevity of the material.

## 5. Conclusions

According to the results and within the limits of this in vitro study, the following conclusions can be drawn:

- irrespective of preparation geometry, the manufacturing processes needed to obtain different marginal finish lines (deep-chamfer, slight-chamfer, feather-edge) did not generate the phenomenon of “transformation toughening” either in the load application areas or at the margins of the restorations in the analyzed brands of zirconia;
- after wear-simulation of one year of chewing (fatigue cyclic load), monoclinic zirconia was not found either on the top or at the margins of the copings;
- manufacturing milling, even in thin thickness, did not cause any structural modification of zirconia ceramics “as received by manufacturers” both before and after chewing simulation;
- further laboratory studies and RCTs are needed to investigate if longer chewing time can produce negative effects on zirconia.

## Figures and Tables

**Table 1:** Tested materials divided in groups, their compositions, and marginal preparations. All the groups were tested as received by manufacturers and after simulated artificial chewing for 1 year.

Group	Material	Composition	Preparation
G1a (n = 10)	NobelProcera Zirconia (Nobel Biocare)	ZrO_2_ + Y_2_O_3_ + HfO_2_ > 99%, Y_2_O_3_ 4.5-5.4%, HfO_2_ < 5%, Al_2_O_3_ < 0.5%	Feather-edge
G1b (n = 10)			Slight-chamfer
G1c (n = 10)			Deep-chamfer
G2a (n = 10)	LAVA Classic (3M ESPE)	3 mol% Y-TZP + Al_2_O_3_	Feather-edge
G2b (n = 10)			Slight-chamfer
G2c (n = 10)			Deep-chamfer
G3a (n = 10)	LAVA Plus (3M ESPE)	3 mol% Y-TZP + Al_2_O_3_ 0.1% + ionic staining components	Feather-edge
G3b (n = 10)			Slight-chamfer
G3c (n = 10)			Deep-chamfer

## References

[1] Vigolo P., Mutinelli S. (2012). Evaluation of zirconium-oxide-based ceramic single-unit posterior fixed dental prostheses (FDPs) generated with two CAD/CAM systems compared to porcelain-fused-to-metal single-unit posterior FDPs: A 5-year clinical prospective study. J. Prosthodont..

[2] Zarone F., Russo S., Sorrentino R. (2011). From porcelain-fused-to-metal to zirconia: Clinical and experimental considerations. Dent. Mater..

[3] Sorrentino R., De Simone G., Tetè S., Russo S., Zarone F. (2012). Five-year prospective clinical study of posterior three-unit zirconia-based fixed dental prostheses. Clin. Oral Investig..

[4] Iwai T., Komine F., Kobayashi K., Saito A., Matsumura H. (2008). Influence of convergence angle and cement space on adaptation of zirconium dioxide ceramic copings. Acta Odontol. Scand..

[5] Kokubo Y., Tsumita M., Kano T., Sakurai S., Fukushima S. (2011). Clinical marginal and internal gaps of zirconia all-ceramic crowns. J. Prosthodont. Res..

[6] Ferrari M., Vichi A., Zarone F. (2015). Zirconia abutments and restorations: From laboratory to clinical investigations. Dent. Mater..

[7] Fuzzi M., Tricarico M.G., Ferrari Cagidiaco E., Bonadeo G., Sorrentino R., Ferrari M. (2017). Nanoleakage and internal adaptation of zirconia and lithium disilicate single crowns with knife edge preparation. J. Osseointegr..

[8] Sorrentino R., Triulzio C., Tricarico M.G., Bonadeo G., Gherlone E.F., Ferrari M. (2016). In vitro analysis of the fracture resistance of CAD-CAM monolithic zirconia molar crowns with different occlusal thickness. J. Mech. Behav. Biomed. Mater..

[9] Kisi E.H., Howard C.J. (1998). Crystal structures of zirconia phases and their inter-relation. Key Eng. Mater..

[10] Camposilvan E., Leone R., Gremillard L., Sorrentino R., Zarone F., Ferrari M., Chevalier J. (2018). Aging resistance, mechanical properties and translucency of different yttria-stabilized zirconia ceramics for monolithic dental crown applications. Dent. Mater..

[11] Subarrao E.C., Heuer A.H., Hobbs L.W. (1981). Zirconia: An overview. Science and Technology of Zirconia.

[12] Fabbri G., Fradeani M., Dellificorelli G., De Lorenzi M., Zarone F., Sorrentino R. (2017). Clinical evaluation of the influence of connection type and restoration height on the reliability of zirconia abutments: A retrospective study on 965 abutments with a mean 6-year follow-up. Int. J. Periodontics Restor. Dent..

[13] Comlekoglu M., Dundar M., Ozcan M., Gungor M., Gokce B., Artunc C. (2009). Influence of cervical finish line type on the marginal adaptation of zirconia ceramic crowns. Oper. Dent..

[14] Patroni S., Chiodera G., Caliceti C., Ferrari P. (2010). CAD/CAM technology and zirconium oxide with feather-edge marginal preparation. Eur. J. Esthet. Dent..

[15] Reich S., Petschelt A., Lohbauer U. (2008). The effect of finish line preparation and layer thickness on the failure load and fractography of ZrO2 copings. J. Prosthet. Dent..

[16] Tinschert J., Schulze K.A., Natt G., Latzke P., Heussen N., Spiekermann H. (2008). Clinical behavior of zirconia-based fixed partial dentures made of DC-Zirkon: 3-year results. Int. J. Prosthodont..

[17] Balkaya M.C., Cinar A., Pamuk S. (2005). Influence of firing cycles on the margin distortion of 3 all-ceramic crown systems. J. Prosthet. Dent..

[18] Poggio C.E., Dosoli R., Ercoli C. (2012). A retrospective analysis of 102 zirconia single crowns with knife-edge margins. J. Prosthet. Dent..

[19] Heintze S.D., Rousson V. (2010). Survival of zirconia- and metal-supported fixed dental prostheses: A systematic review. J. Prosthet. Dent..

[20] Guess P.C., Bonfante E.A., Silva N.R., Coelho P.G., Thompson V.P. (2013). Effect of core design and veneering technique on damage and reliability of Y-TZP-supported crowns. Dent. Mater..

[21] Scherrer S.S., Quinn J.B., Quinn G.D., Wiskott H.W. (2007). Fractographic ceramic failure analysis using the replica technique. Dent. Mater..

[22] Lohbauer U., Amberger G., Quinn G.D., Scherrer S.S. (2010). Fractographic analysis of a dental zirconia framework: A case study on design issues. J. Mech. Behav. Biomed. Mater..

[23] Potiket N., Chiche G., Finger I.M. (2004). In vitro fracture strength of teeth restored with different all-ceramic crown systems. J. Prosthet. Dent..

[24] Bindl A., Lüthy H., Mörmann W.H. (2006). Thin-wall ceramic CAD/CAM crown copings: Strength and fracture pattern. J. Oral Rehabil..

[25] Rekow E.D., Silva N.R., Coelho P.G., Zhang Y., Guess P., Thompson V.P. (2011). Performance of dental ceramics: Challenges for improvements. J. Dent. Res..

[26] Aboushelib M.N. (2012). Fatigue and fracture resistance of zirconia crowns prepared with different finish line designs. J. Prosthodont..

[27] Sergo V., Lughi V., Pezzotti G., Lucchini E., Meriani S., Muraki N., Katagiri G., Lo Casto S., Nishida T. (1998). The effect of wear on the tetragonal-to-monoclinic transformation and the residual stress distribution in zirconia-toughened alumina cutting tools. Wear.

[28] Dauskardt R.H., Marshall D.B., Ritchie R.O. (1990). Cyclic fatigue-crack propagation in magnesia-partially stabilized zirconia ceramics. J. Am. Ceram. Soc..

[29] Pezzotti G., Porporati A.A. (2004). Raman spectroscopic analysis of phase-transformation and stress patterns in zirconia hip joints. J. Biomed. Opt..

[30] Naumenko A.P., Berezovska N.I., Biliy M.M., Shevchenko O.V. (2008). Vibrational Analysis and Raman Spectra of Tetragonal Zirconia. Phys. Chem. Solid State.

[31] Lughi V., Sergo V. (2010). Low temperature degradation -aging- of zirconia: A critical review of the relevant aspects in dentistry. Dent. Mater..

[32] Ramos C.M., Tabata A.S., Cesar P.F., Rubo J.H., Fracisconi P.A., Sanches Borges A.F. (2015). Application of Micro-Raman Spectroscopy to the Study of Yttria-Stabilized Tetragonal Zirconia Polycrystal (Y-TZP) Phase Transformation. Appl. Spectrosc..

[33] Cadenaro M., Codan B., Navarra C.O., Marchesi G., Turco G., Di Lenarda R., Breschi L. (2011). Contraction stress, elastic modulus, and degree of conversion of three flowable composites. Eur. J. Oral Sci..

[34] Navarra C.O., Cadenaro M., Armstrong S.R., Jessop J., Antoniolli F., Sergo V., Di Lenarda R., Breschi L. (2009). Degree of conversion of Filtek Silorane Adhesive System and Clearfil SE Bond within the hybrid and adhesive layer: An in situ Raman analysis. Dent. Mater..

[35] Beuer F., Stimmelmayr M., Gueth J.F., Edelhoff D., Naumann M. (2012). In vitro performance of full-contour zirconia single crowns. Dent. Mater..

[36] Schmitter M., Mueller D., Rues S. (2012). Chipping behaviour of all-ceramic crowns with zirconia framework and CAD/CAM manufactured veneer. J. Dent..

[37] Stawarczyk B., Ozcan M., Hallmann L., Roos M., Trottmann A., Hämmerle C.H. (2012). Impact of air-abrasion on fracture load and failure type of veneered anterior Y-TZP crowns before and after chewing simulation. J. Biomed. Mater. Res. B Appl. Biomater..

[38] Nelson J. (2014). Wheeler’s Dental Anatomy, Physiology and Occlusion.

[39] Trier A.C., Parker M.H., Cameron S.M., Brousseau J.S. (1998). Evaluation of resistance form of dislodged crowns and retainers. J. Prosthet. Dent..

[40] Kokubo Y., Tsumita M., Kano T., Fukushima S. (2011). The influence of zirconia coping designs on the fracture load of all-ceramic molar crowns. Dent. Mater. J..

[41] Rosentritt M., Siavikis G., Behr M., Kolbeck C., Handel G. (2008). Approach for valuating the significance of laboratory simulation. J. Dent..

[42] Rosentritt M., Behr M., van der Zel J.M., Feilzer A.J. (2009). Approach for valuating the influence of laboratory simulation. Dent. Mater..

[43] Lutz F., Krejci I., Barbakow F. (1992). Chewing pressure vs. wear of composites and opposing enamel cusps. J. Dent. Res..

[44] Steiner M., Mitsias M.E., Ludwig K., Kern M. (2009). In vitro evaluation of a mechanical testing chewing simulator. Dent. Mater..

[45] Inokoshi M., Shimizu H., Nozaki K., Takagaki T., Yoshihara K., Nagaoka N., Zhang F., Vleugels J., Van Meerbeek B., Minakuchi S. (2018). Crystallographic and morphological analysis of sandblasted highly translucent dental zirconia. Dent. Mater..

[46] Roitero E., Anglada M., Mücklich F., Jiménez-Piqué E. (2018). Mechanical reliability of dental grade zirconia after laser patterning. J. Mech. Behav. Biomed. Mater..

[47] Rohr N., Märtin S., Fischer J. (2018). Correlations between fracture load of zirconia implant supported single crowns and mechanical properties of restorative material and cement. Dent. Mater. J..

[48] Poggio C., Pigozzo M., Ceci M., Scribante A., Beltrami R., Chiesa M. (2016). Influence of different luting protocols on shear bond strength of computer aided design/computer aided manufacturing resin nanoceramic material to dentin. Dent. Res. J..

[49] Li J., Wang X., Lin Y., Deng X., Li M., Nan C. (2016). In Vitro Cell Proliferation and Mechanical Behaviors Observed in Porous Zirconia Ceramics. Materials.

[50] Volpato C.A.M., Carvalho Ó.S.N., Pereira M.R.D.C., Correia Pereira da Silva F.S. (2019). Evaluation of the color and translucency of glass-infiltrated zirconia based on the concept of functionally graded materials. J. Prosthet. Dent..

[51] Kelly J.R., Denry I. (2008). Stabilized zirconia as a structural ceramic: An overview. Dent. Mater..

[52] Kosmac T., Oblak C., Jevnikar P., Funduk N., Marion L. (2000). Strength and reliability of surface treated Y-TZP dental ceramics. J. Biomed. Mater. Res..

[53] Bal B.S., Zhu W., Zanocco M., Marin E., Sugano N., McEntire B.J., Pezzotti G. (2017). Reconciling in vivo and in vitro kinetics of the polymorphic transformation in zirconia-toughened alumina for hip joints: I. Phenomenology. Mater. Sci. Eng. C Mater. Biol. Appl..

[54] Sundh A., Molin M., Sjögren G. (2005). Fracture resistance of yttrium oxide partially-stabilized zirconia all-ceramic bridges after veneering and mechanical fatigue testing. Dent. Mater..

[55] Swab J.J. (1991). Low temperature degradation of Y-TZP materials. J. Mater. Sci..

[56] Cotes C., Arata A., Melo R.M., Bottino M.A., Machado J.P.B., Souza R.O.A. (2014). Effects of aging procedures on the topographic surface, structural stability, and mechanical strength of a ZrO_2_-based dental ceramic. Dent. Mater..

[57] Chevalier J., Gremillard L., Deville S. (2007). Low-temperature degradation of zirconia and implications for biomedical implants. Annu. Rev. Mater. Res..

[58] Turrell G., Turrell G., Corset J. (1996). The Raman effect. Raman Microscopy.

[59] Pereira G.K., Venturini A.B., Silvestri T., Dapieve K.S., Montagner A.F., Soares F.Z., Valandro L.F. (2015). Low-temperature degradation of Y-TZP ceramics: A systematic review and meta-analysis. J. Mech. Behav. Biomed. Mater..

[60] Komine F., Iwai T., Kobayashi K., Matsumura H. (2007). Marginal and internal adaptation of zirconium dioxide ceramic copings and crowns with different finish line designs. Dent. Mater. J..

[61] Beuer F., Aggstaller H., Richter J., Edelhoff D., Gernet W. (2009). Influence of preparation angle on marginal and internal fit of CAD/CAM-fabricated zirconia crown copings. Quintessence Int..

[62] Lohbauer U., Petschelt A., Greil P. (2002). Lifetime prediction of CAD/CAM dental ceramics. J. Biomed. Mater. Res..

[63] Di Febo G., Carnevale G., Sterrantino S.F. (1985). Treatment of a case of advanced periodontitis: Clinical procedures utilizing the “combined preparation” technique. Int. J. Periodontics Restor. Dent..

[64] Beuer F., Aggstaller H., Edelhoff D., Gernet W. (2008). Effect of preparation design on the fracture resistance of zirconia crown copings. Dent. Mater. J..

[65] Proos K.A., Swain M.V., Ironside J., Steven G.P. (2003). Influence of core thickness on a restored crown of a first premolar using finite element analysis. Int. J. Prosthodont..

[66] Cho L.R., Choi J., Yi Y.J., Park C.J. (2004). Effect of finish line variants on marginal accuracy and fracture strength of ceramic optimized polymer/fiber-reinforced composite crowns. J. Prosthet. Dent..

[67] Ramos G.F., Monteiro E.B., Bottino M.A., Zhang Y., de Melo R.M. (2015). Failure Probability of Three Designs of Zirconia Crowns. Int. J. Periodontics Restor. Dent..

